# Relative cost and outcomes in the intensive care unit of acute lung injury (ALI) due to pandemic influenza compared with other etiologies: a single-center study

**DOI:** 10.1186/2110-5820-2-41

**Published:** 2012-08-28

**Authors:** Jonathan Wiesen, John J Komara, Esteban Walker, Herbert P Wiedemann, Jorge A Guzman

**Affiliations:** 1 Resident, Internal Medicine, Cleveland Clinic Foundation, Cleveland, USA; 2 Department of Critical Care, Cleveland Clinic Foundation, Cleveland, USA; 3 Department of Quantitative Health Sciences, Cleveland Clinic Foundation, Cleveland, USA; 4 Respiratory Institute, Cleveland Clinic Foundation, Cleveland, USA; 5 Medical Intensive Care Unit, Cleveland Clinic Foundation, Cleveland, USA; 6 Respiratory Institute, A909500 Euclid Avenue, Cleveland, OH, 44195, USA

**Keywords:** ARDS, ALI, Influenza A, Novel influenza, Mechanical ventilation, Hospital cost

## Abstract

**Background:**

Critical illness due to 2009 H1N1 influenza has been characterized by respiratory complications, including acute lung injury (ALI) or acute respiratory distress syndrome (ARDS), and associated with high mortality. We studied the severity, outcomes, and hospital charges of patients with ALI/ARDS secondary to pandemic influenza A infection compared with ALI and ARDS from other etiologies.

**Methods:**

A retrospective review was conducted that included patients admitted to the Cleveland Clinic MICU with ALI/ARDS and confirmed influenza A infection, and all patients admitted with ALI/ARDS from any other etiology from September 2009 to March 2010. An itemized list of individual hospital charges was obtained for each patient from the hospital billing office and organized by billing code into a database. Continuous data that were normally distributed are presented as the mean ± SD and were analyzed by the Student’s *t* test. The chi-square and Fisher exact tests were used to evaluate differences in proportions between patient subgroups. Data that were not normally distributed were compared with the Wilcoxon rank-sum test.

**Results:**

Forty-five patients were studied: 23 in the H1N1 group and 22 in the noninfluenza group. Mean ± SD age was similar (44 ± 13 and 51 ± 17 years, respectively, *p* = 0.15). H1N1 patients had lower APACHE III scores (66 ± 20 vs. 89 ± 32, *p* = 0.015) and had higher Pplat and PEEP on days 1, 3, and 14. Hospital and ICU length of stay and duration of mechanical ventilation were comparable. SOFA scores over the first 2 weeks in the ICU indicate more severe organ failure in the noninfluenza group (*p* = 0.017). Hospital mortality was significantly higher in the noninfluenza group (77 vs. 39%, *p* = 0.016). The noninfluenza group tended to have higher overall charges, including significantly higher cost of blood products in the ICU.

**Conclusions:**

ALI/ARDS secondary to pandemic influenza infection is associated with more severe respiratory compromise but has lower overall acuity and better survival rates than ALI/ARDS due to other causes. Higher absolute charges in the noninfluenza group are likely due to underlying comorbid medical conditions.

## Background

The spread of a novel H1N1 strain of the Influenza A virus represents the first pandemic of the 21^st^ century and the first influenza pandemic since 1968 [1]. Compared with seasonal influenza, this strain was more prevalent in younger-aged individuals, obese patients, and pregnant women [2-12]. Severe cases of pandemic H1N1 resulted in respiratory failure thought to be secondary to direct cell damage and systemic cytokine release resulting in acute lung injury (ALI) or acute respiratory distress syndrome (ARDS) requiring prolonged ventilatory assistance and the frequent use of rescue therapies [4,5,8,13-17].

Limited data exist that compare the clinical differences between ALI in H1N1 patients and ALI arising from other etiologies. Furthermore, whereas a number of studies have assessed different aspects of the economic impact of the recent pandemic [18-21], few have focused on the health care cost of the pandemic, particularly the utilization of limited ICU resources.

We report the severity, clinical outcomes, and hospital charges of ALI/ARDS secondary to pandemic influenza A infection compared with ALI/ARDS from other etiologies during a similar period of time. Based on clinical bedside observations and published reports [4,5,8], we hypothesize that ALI/ARDS secondary to pandemic influenza is associated with similar ICU outcomes but increased resource utilization and higher hospital charges due to the frequent need for rescue interventions and prolonged ventilatory assistance.

## Methods

The study was approved by the Human Investigation Committee of the Cleveland Clinic Foundation (CCF) (Institutional Review Board approval # 10–229) as a retrospective, single-center study at the CCF Medical ICU.

Patients were identified from a unit-based acute lung injury screening database (Cleveland Clinic is one of the centers participating in the ARDSnetwork) and the H1N1 patient log maintained during the fall-winter season of 2009–2010. Patients were included if they met criteria for ALI (PaO_2_/FiO_2_ ≤300; acute bilateral infiltrates; positive pressure ventilation via endotracheal tube; and no clinical evidence of left atrial hypertension or congestive heart failure) between the months of September 2009 to March 2010—the time that influenza infection was most prevalent. Diagnostic methods for influenza A virus detection consisted of rapid antigen testing, polymerase chain reaction (rtPCR), and viral culture from nasopharyngeal swabs, tracheal aspirates, and bronchioalveolar lavage specimens. The patients were grouped into two categories: those with laboratory-proven H1N1 infection; and those in whom H1N1was not clinically suspected. Only patients with confirmed infection were included in the influenza group to ensure that the clinical course of the disease was accurately captured. Patients were excluded from the study if they did not meet the above criteria for ARDS, or if clinical suspicion pointed to a likely pandemic viral infection with negative diagnostics.

A Research Electronic Data Capture (REDCap) database was constructed with a complete listing of the patient’s demographic and clinical information, including age, gender, height, weight, body mass index (BMI), presenting symptoms, past medical history, primary reason for admission to the ICU, vital signs, presence of vasopressors, laboratory values, ventilator settings and respiratory parameters, Acute Physiology and Chronic Health Evaluation (APACHE) III and Sequential Organ Failure Assessment (SOFA) scores on admission to the MICU, number of intubated days, duration of ICU and hospital stay, mortality, and rescue therapies (namely inhaled nitric oxide, proning, high-frequency oscillatory ventilation, and extracorporeal membrane oxygenation [ECMO]) [22]. The data collection was de-identified and collected in accordance with HIPAA guidelines.

As part of the routine MICU respiratory therapy protocol, mechanical ventilation parameters are recorded every 4 hours. All patients are managed according to a mechanical ventilation protocol that incorporates the use of nonconventional modes when a lung protective strategy on conventional modes failed to provide adequate oxygenation. The following criteria were used to define the analyzed parameters: 1) mode of ventilation: the mode of ventilation that was used for the longest time for a given day; 2) PaO_2_/FiO_2_: worst daily ratios were recorded; 3) plateau pressure (Pplat): for patients on volume control ventilation the airway pressure was measured after a 5-second inspiratory hold without concomitant active inspiratory efforts, and for patients on pressure control ventilation (PCV) the highest total system pressure (PEEP + inspiratory pressure) was recorded; 4) positive end expiratory pressure (PEEP): the value corresponding to the highest PEEP for the day was recorded; 5) tidal volume (Vt): the largest daily volume was recorded. Respiratory data were captured on the first day of intubation (day 1) and then on subsequent days 3, 7, and 14 of mechanical ventilation. There were no differences in ventilator protocols or management between the two groups.

An itemized bill of individual charges for each patient was obtained from the hospital billing office and was organized by billing code into the following categories: room/board, pharmacy, supplies, laboratory, radiology, surgical (including procedures performed under general anesthesia), blood products, respiratory services, dialysis, and miscellaneous (which included some professional fees, nonsurgical procedures and phlebotomy, and diagnostics not included in the other categories, such as electroencephalograms, electrocardiograms, echocardiograms, cardiac catheterizations, and vascular studies). The values represent the hospital charges for the aforementioned services rather than the actual reimbursement, which may be subject to more variability. The single-center nature of the study removes interfacility differences in clinical and billing practices.

Continuous data that were normally distributed are presented as the mean ± SD and were analyzed by the Student’s *t* test. The chi-square and Fisher exact tests were used to evaluate differences in proportions between patient groups. In instances where the data were not normally distributed, the groups were compared with the Wilcoxon rank-sum test. Differences were considered statistically significant if the p value was <0.05.

## Results

Fifty-one patients were identified in the acute lung injury screening database between September 2009 and March 2010. Twenty-two met criteria for ALI and did not have confirmed or suspected H1N1 infection and were thus included in the noninfluenza group (ALI/ARDS secondary to noninfluenza etiologies). Thirty-six patients in the H1N1 patient log had confirmed influenza A testing. Of those, 23 had ALI requiring mechanical ventilation (MV) during their MICU stay and were included in our analysis.

Demographics, presenting symptoms, past medical history, and acuity on admission are shown in Table 1. Patients in the influenza group tended to be younger with a higher BMI. Patients in the influenza group presented more often with lower respiratory infection (100 vs. 73%, *p* = 0.135) and had increased requirement for mechanical ventilation on admission to the ICU (96 vs. 68%, *p* = 0.022). On the other hand, the noninfluenza group had a higher propensity to present with shock requiring vasopressors (45 vs. 22%, respectively, *p* = 0.07). The primary cause of ALI in the H1N1 group was pneumonia (n = 23), whereas in the noninfluenza group the etiologies were more varied, including pneumonia (n = 9), sepsis (n = 5), aspiration of gastric contents (n = 2), transfusion reaction (n = 1), and other (n = 5). Whereas seven patients (30%) in the H1N1 group were considered healthy, only one patient (5%) in the noninfluenza group had no comorbid medical conditions on admission to the ICU (Table 1). This difference is reflected in the lower mean APACHE III score on admission to the ICU in the H1N1 group (66 ± 20 vs. 89 ± 32, *p* = 0.015), despite similar SOFA scores (8.3 ± 3.4 and 9.2 ± 4.1, *p* = 0.44).

**Table 1:** **Demographics, comorbid medical conditions, presenting symptoms, vital signs, laboratory values, and acuity scores on ICU admission**^**a**^

**Baseline characteristics**	**Influenza (n = 23)**	**Noninfluenza (n = 22)**	***p*value**^**b**^
Age (yr)	44 ± 13	51 ± 17	0.15
Gender (M:F)	10:13	11:11	0.77
BMI (kg/m^2^)	40 ± 22	32 ± 12	0.15
**Comorbid medical conditions**			
Hypertension	13	12	0.57
Tobacco use	12	12	0.68
Chronic renal insufficiency	1	6	0.05
Malignancy	2	7	0.07
Immunosuppression	4	6	0.49
Alcohol abuse	1	6	0.05
Ischemic heart disease	1	4	0.19
Cirrhosis	0	4	0.05
Prior pulmonary disease	4	7	0.31
**Presenting symptoms to ICU**			
Lower respiratory infection	100% (23)	73% (16)	0.135
CNS infection	0	4.5% (1)	0.489
Shock requiring vasopressors	22% (5)	45% (10)	0.07
**Vital Signs**			
SBP (mm Hg)	131 ± 23	110 ± 19	0.004
DBP (mm Hg)	71.6 ±12	60.5 ± 10	0.004
Temperature (°C)	37.8 ± 1.5	37 ± 1.2	0.089
RR (breath/min)	23.4 ± 8.7	31.7 ± 21.4	0.14
HR (beats/min)	106 ± 27.3	108 ± 18	0.81
**Laboratory values**			
WBC (k/μL)	10.6 ±7.46	11.4 ± 10.7	0.77
Platelets (k/μL)	196 ± 119	138 ± 127	0.12
Creatinine (mg/dL)	1.59 ± 1.13	1.62 ± 1.22	0.92
Bilirubin (mg/dL)	0.65 ± 0.46	3.3 ± 8.3	0.13
CK (U/L)	1155 ± 1642	723 ± 1551	0.45
**Cause of ALI**			
Pneumonia	100% (23)	41% (9)	<0.001
Sepsis	0	23% (5)	0.02
Aspiration	0	9% (2)	0.23
Transfusion reaction	0	5% (1)	0.49
Other	0	23% (5)	0.02
**Acuity on ICU admission**			
APACHE III score	66 ± 20 (19)	89 ± 32 (20)	0.015
SOFA score	8.3 ± 3.4	9.2 ± 4.1	0.44
**Length of stay (median ± IQR)**			
ICU length of stay (days)	16 ± 22	24.5 ± 26.5	0.17^c^
Hospital length of stay (days)	12 ± 15	17 ± 25.5	0.45^c^

^a^Values expressed as mean ± SD unless otherwise noted; ^b^*t* test or chi-squared; ^c^Wilcoxon test. *CNS*, central nervous system; *SBP*, systolic blood pressure; *DBP*, diastolic blood pressure; *RR*, respiratory rate; *HR*, heart rate; *WBC*, white blood cell; *CK*, creatinine kinase; *APACHE*, Acute Physiology and Chronic Health Evaluation; *SOFA*, Sequential Organ Failure Assessment.

There were no statistically significant differences between the two groups for initial laboratory data, including white blood cell count, platelets, serum creatinine, bilirubin, and creatinine kinase. The number of patients who developed acute renal failure that required dialysis throughout their ICU stay was the same (n = 8) in both groups. SOFA scores on days 1, 3, 7, and 14 of mechanical ventilation indicate that patients in the noninfluenza group had more severe organ failure during their ICU stay (*p* = 0.017; Table 2).

**Table 2:** **SOFA scores on days 1, 3, 7, and 14 of mechanical ventilation**^**a**^

	**Influenza (n = 23)**	**Noninfluenza (n = 22)**	***p*value**
Day 1	8.8 ± 3.8	12.4 ± 3.8	0.003
	(23)	(22)	
Day 3	8.7 ± 4.3	11.2 ± 4.6	0.07
	(23)	(21)	
Day 7	9.0 ± 4.3	12.4 ± 4.3	0.01
	(21)	(17)	
Day 14	7.6 ± 6.4	9.3 ± 6.1	0.37
	(11)	(14)	

^**a**^All values expressed as mean ± SD. Using mixed models, the overall *p* value comparing the influenza and noninfluenza groups is 0.017. The trend over time was not significant (*p* = 0.1).

Table 3 shows oxygenation index and mechanical ventilation related parameters on days 1, 3, 7, and 14. There was a nonsignificant trend toward worsening hypoxia in the H1N1 group, despite significantly higher PEEP and Pplat on days 1, 3, and 14. Tidal volumes were comparable throughout. Plateau pressures in the H1N1 group were high due to the relative decrease in pulmonary compliance in H1N1-related lung injury. Four patients in both groups were ventilated with airway pressure release ventilation (APRV). More patients in the influenza group required rescue therapies on day 1 of mechanical ventilation (4 vs. 0, respectively, *p* = 0.108); however, similar numbers of patients in both groups required rescue therapies over the duration of MV (7 and 5 patients, respectively). Rescue therapies in the H1N1 group included inhaled NO (n = 4), ECMO (n = 2), prone ventilation (n = 3), and high-frequency ventilation (n = 1), and in the noninfluenza group only inhaled NO (n = 3) and prone ventilation (n = 2).

**Table 3:** Oxygenation indices and mechanical ventilation parameters on days 1, 3, 7, and 14

	**Day 1 of mechanical ventilation**		**Day 3 of mechanical ventilation**		**Day 7 of mechanical ventilation**		**Day 14 of mechanical ventilation**	
	**Influenza (n = 23 )**	**Non influenza (n = 22 )**	**Influenza (n = 23 )**	**Non influenza (n = 21 )**	**Influenza (n = 21 )**	**Non influenza (n = 17 )**	**Influenza (n = 11)**	**Non influenza (n = 13)**
PaO_2/_FiO_2_	150 ± 107	156 ± 95	181 ± 82	206 ± 80.2	185 ± 96	230 ± 118	148 ± 69	251 ± 146
P_Plat_ (cm H_2_O)	37 ± 8	25 ± 6^a^	34 ± 7	24 ± 7^a^	31 ± 10	25 ± 9	38 ± 9	24 ± 4^a^
PEEP (cm H_2_O)	16 ± 6	9.3 ± 6^a^	15 ± 7	9.2 ± 6^a^	13 ± 6.4	9.5 ± 6.4	14 ± 7.4	8 ± 4.3^a^
Vt (mL/kg)	7.7 ± 1.4	7.4 ± 1.1	8.5 ± 2	7.6 ± 1	8 ± 2	8 ± 2.6	7.7 ± 1.8	8 ± 1.8

^a^*p* < 0.05 with *t* test.

**Figure 1:**
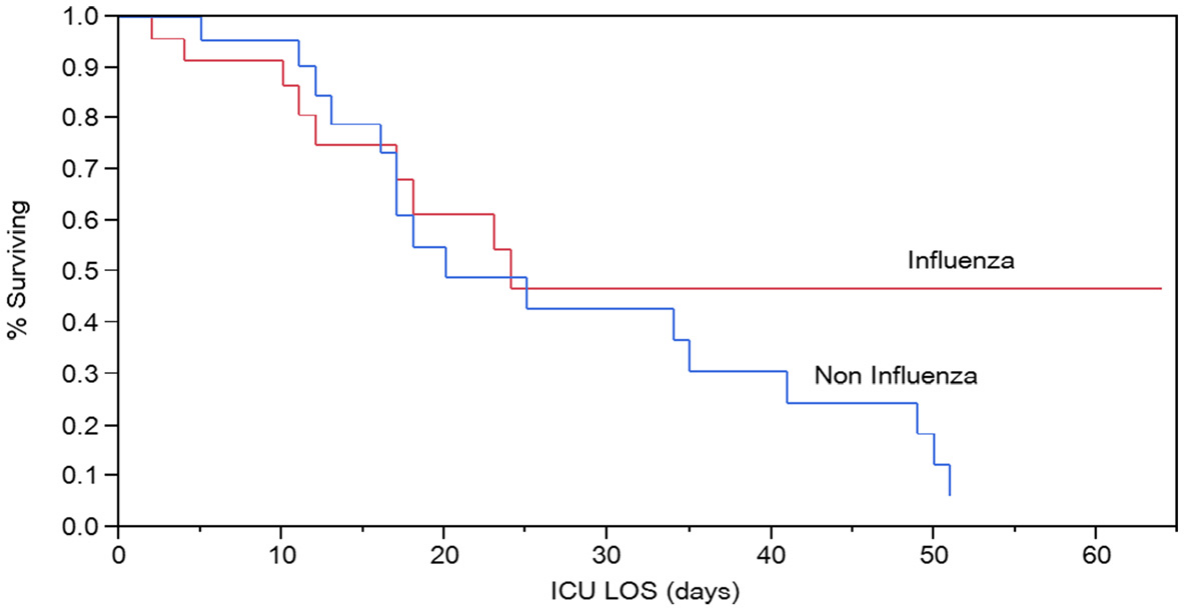
**Kaplan-Meier curve of ICU mortality (log rank, *p *** **= 0.26).**

Mechanical ventilation days were comparable between groups (22 ± 17 vs. 19 ± 15 days for groups I and II, respectively, *p* = 0.53) as were 28-day ventilator-free days (5 ± 7.6 and 4.6 ± 9, *p* = 0.88). Four patients in the H1N1 group and seven in the noninfluenza group underwent a tracheostomy procedure. Hospital and ICU LOS were comparable (median ± IQR: 16 ± 22 vs. 24.5 ± 26.5 and 12 ± 15 vs. 17 ± 25.5 days for the influenza group and II, respectively, Wilcoxon *p* = 0.17 and 0.45). Mortality was significantly higher for patients in the noninfluenza group (77 vs. 39%, *p* = 0.016). Interestingly, a Kaplan-Meier curve of ICU mortality (Figure 1) indicates that patients in the H1N1 group were more likely to be discharged alive from the ICU when the length of stay was greater than 25 days, despite a trend toward higher mortality within the first 2 weeks.

**Figure 2:**
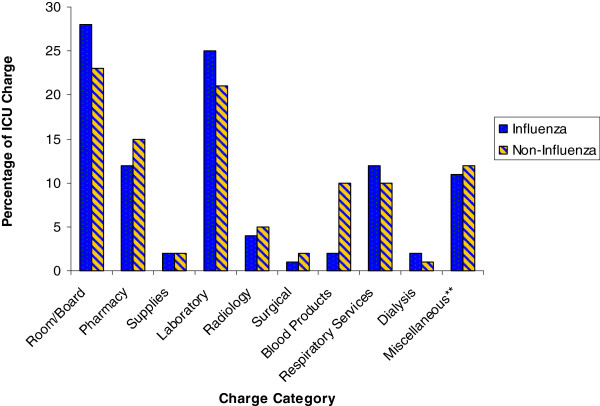
**Percentage of ICU charges by category.** **Miscellaneous includes professional fees, nonsurgical procedures and phlebotomy, and diagnostics not included in the other categories, such as electroencephalograms, electrocardiograms, echocardiograms, cardiac catheterizations, and vascular studies.

Even though all charges were higher in the noninfluenza group, only the difference in blood products utilized in the ICU was significant (4 ± 6 vs. 21 ± 25 thousands of U.S. dollars, Wilcoxon *p* < 0.001; Table 4). Differences in ICU charges in pharmacy (*p* = 0.23), supplies (*p* = 0.09), radiology (*p* = 0.08), and miscellaneous (*p* = 0.09) were large but not significant due to considerable variation. The proportion of charges in each of the major categories was similar between the groups (Figure 2). The average total ICU cost per patient (253 ± 193 vs. 350 ± 270 thousands of U.S. dollars, Wilcoxon *p* = 0.19) and the average ICU cost per patient per day (13 ± 4 vs. 15 ± 6 thousands of U.S. dollars, Wilcoxon *p* = 0.06) tended to be higher in the noninfluenza group.

**Table 4:** Mean hospital charges per patient (in thousands of U.S. dollars)

	**ICU charges**		**Non ICU charges**		**Total hospital charges**	
	**Influenza (n = 23 )**	**Non influenza (n = 22 )**	**Influenza (n = 11 )**	**Non influenza (n = 13 )**	**Influenza (n = 23 )**	**Non influenza (n = 22 )**
Room/board	71 ± 53	81 ± 62	15 ± 30	13 ± 17	86 ± 65	94 ± 69
Pharmacy	29 ± 35	54 ± 74	12 ± 31	16 ± 34	41 ± 49	70 ± 92
Supplies	4 ± 4	6 ± 7	1 ± 3	1 ± 2	5 ± 6.5	8 ± 7
Laboratory	63 ± 50	74 ± 55	27 ± 82	12 ± 10	90 ± 99	86 ± 58
Radiology	10 ± 9	17 ± 15	2 ± 4	5 ± 5	12 ± 10	22 ± 17
Surgical	3 ± 5	5 ± 10	6 ± 15	1 ± 3	9 ± 18	7 ± 11
Blood products	4 ± 6	21 ± 25^c^	4 ± 11	5 ± 12	8 ± 15	27 ± 34^c^
Respiratory Services	31 ± 22	36 ± 28	7 ± 11	5 ± 5	37 ± 25	40 ± 29
Dialysis	4 ± 8	4 ± 6	2 ± 7	.1 ± 0.35	6 ± 13	4 ± 6
Miscellaneous^a^	35 ± 26	50 ± 36	15 ± 32	12 ± 12	50 ± 50	61 ± 40
Total charges^b^						
Mean (95% CI)	253 (169, 336)	350 (213, 468)	89 (0, 184)	70 (32, 107)	342 (203, 481)	419 (282, 556)
Median [IQR]	156 [124–392]	252 [182–461]	23 [13–54]	52 [11–91]	195 [148–503]	304 [203–686]

^a^Miscellaneous includes professional fees, nonsurgical procedures and phlebotomy, and diagnostics not included in the other categories, such as electroencephalograms, electrocardiograms, echocardiograms, cardiac catheterizations, and vascular studies. ^b^per patient. ^c^*p* <0.05 with Wilcoxon test.

## Discussion

The fall of 2009 heralded the influx of patients suffering from severe hypoxic respiratory complications secondary to the pandemic H1N1 influenza to ICUs across the country. Due to the severity of pulmonary disease that many of these patients experienced, perception among treating clinicians was that these patients would have worse outcomes and consume more resources, as measured by hospital charges, than patients who developed ALI from other etiologies. We demonstrated that, contrary to what was perceived, pandemic influenza A ALI/ARDS was associated with a lower acuity and, consequently, lower hospital mortality that ALI/ARDS from other etiologies, and had a similar ICU and hospital LOS. ICU and total hospital charges reflected a trend toward higher overall charges for room and board, blood products, pharmacy, and overall charge per patient in the noninfluenza group.

In accordance with other descriptive reports of pandemic influenza [2-12], patients who tested positive for H1N1 infection, tended to be young (no patients >64 years old), obese (15 had BMI >30 kg/m^2^), and in relatively good health (30% with no comorbid medical conditions). There were no pregnant patients in either group. Compared with other studies of pandemic influenza patients who required mechanical ventilation, SOFA scores (mean 8.3) were similar, although APACHE II (25 ± 9) scores were higher [5-8,14,16,17,23]. The degree of respiratory compromise in our patients was more severe than other reports judging by the higher PEEP requirements and longer duration of mechanical ventilation, which was roughly double that reported in other studies [4-6,8,11,13,14,16,23]. Plateau pressures in these studies were not consistently reported. However, despite significantly longer ventilation duration and prolonged ICU and hospital stays, the mortality in our cohort was not higher than that seen in other studies, which ranged from 22–41% in patients who required mechanical ventilation [4-6,8,11,13,14,16,23].

Looking at the different patient characteristics between groups, it may be tempting to postulate that the higher rate of patients with pulmonary ARDS in the H1N1 group, in contrast to prevalent nonpulmonary ARDS in the noninfluenza group, would correlate with a higher PEEP response among the latter [24]. Our findings suggest the contrary. Patients in the H1N1 group had higher mean plateau pressure, likely indicative of lower compliance. The similarity of PaO_2_/FiO_2_ ratios in the two groups may be a reflection of higher PEEP values used in the H1N1 group for lung recruitment, rather than being indicative of comparable degrees of lung injury. Although assessing recruitability from this retrospective analysis is difficult and may be inaccurate, the higher PEEP used and the implication of lower compliance observed are predictors of potentially recruitable lung [24]. These observations support the recent call for a reevaluation of the ALI and ARDS criteria to account for this heterogeneity in the patient population [25].

A number of important differences between the two cohorts emerged as well. As expected, the noninfluenza group was older, had more comorbid medical conditions, and less often presented to the ICU with respiratory failure. The degree of ventilator support was significantly higher in the H1N1 group on days 1, 3, and 14, and there was a trend to more severe hypoxemia during that time as well. Nevertheless, the use of use of APRV and rescue therapies was comparable in both groups. Despite more severe respiratory compromise, H1N1 patients did not have longer time on the ventilator, longer ICU or hospital stays, or higher mortality. Although SOFA scores were similar, the noninfluenza group had significantly higher APACHE III scores, likely secondary to points assigned to comorbid medical conditions. The high acuity of illness, as well as the presence of severe comorbidities, such as solid and hematologic oncologic conditions (7 patients), chronic renal insufficiency (6 patients), and cirrhosis of the liver (4 patients), likely contributed to the poor outcomes in the noninfluenza group. Conversely, despite more severe respiratory compromise, patients in the H1N1 group were more likely to recover due to their younger age and better overall health histories.

The 77% mortality in the noninfluenza group was much higher than typically reported in clinical trials, with one notable exception [26]. However, reports from tertiary care centers involving patient cohorts with similar underlying comorbid conditions have reported equally high mortality rates [27]. Our observation brings up an interesting point, namely the difference between the reported mortality in clinical trials and the observed mortality in a similar clinical condition affecting patients that would have been excluded from such trials due to coexisting comorbidities. A Kaplan-Meier plot of ICU mortality (Figure 1) indicates that although patients in the H1N1 group were less likely to survive the first 14 days of ICU care, those that did survive past day 25 were more likely to be discharged alive from the hospital. Patients in the noninfluenza group were unlikely to survive if their ICU length of stay exceeded 3 weeks.

ARDS is among the most expensive conditions encountered in the ICU [28]. In 1984, Bellamy and Oye described the charges of patients with ARDS, with the most expensive being room and board (30%), clinical laboratory (24%), pharmacy (14%), and inhalation therapy and ventilation (8%) [27]. Twenty-five years later, our study indicates that the aforementioned categories continue to represent the most expensive charges incurred by ARDS patients in the ICU.

The overall similarity of charges in room and board and respiratory therapy between the two groups is likely indicative of the comparative durations of hospitalization and mechanical ventilation. Interestingly, despite higher ventilatory requirements and more severe hypoxemia in the H1N1 group, respiratory charges were similar between the two groups, suggesting that the high cost of maintaining a patient on mechanical ventilation is independent of the degree of ventilator support necessary. Thus, respiratory charges are more likely a reflection of duration of mechanical ventilation rather than the degree of ventilator support necessary. Absolute ICU charges for room and board, blood products, pharmacy, radiology, average daily charge, and overall charge per patient were larger in the noninfluenza group. ICU charges for blood products in the noninfluenza group were greater by a factor of four, and pharmacy charges double that of the H1N1 group. This finding is likely a reflection of the higher prevalence of underlying comorbid medical conditions in the noninfluenza group, such as malignancy and cirrhosis, which require expensive medications and predispose to anemia. Moreover, the high mortality in this cohort likely precluded even higher hospital charges. Nevertheless, the H1N1 cohort amassed charges of similar magnitude to the most ill and expensive patients in the ICU, indicating the abundant health care resources consumed by severe pandemic influenza infection.

There are a number of limitations to our study. As a retrospective chart review rather than a prospective investigation, the information was culled from sources that were at times incomplete. Second, the study contained a relatively small number of patients, and measures taken to ensure internal validity of each group, such as limiting the influenza group to confirmed H1N1 infection and the noninfluenza group to the duration of the influenza season, further limited its size. Additionally, whereas our study provides descriptive information relevant to the patient population of our institution and tertiary referral centers with similar acuity, other ICUs may be exposed to a different cohort of patients. On the other hand, as a single-center study, potential differences in clinical and billing practices could be minimized. Although a comprehensive charge profile of each patient was generated, trends in the timing of charges could not be obtained. Finally, the hospital charge data were mined from an extensive database divided by charge coding, and therefore, some charges may have been mislabeled or inappropriately categorized.

## Conclusions

Our study provides interesting observations about the clinical course, outcomes, and cost of the H1N1 influenza pandemic. Although patients with severe pulmonary complications of pandemic influenza infection have poor oxygenation and require significant ventilatory support and rescue therapies, their younger age and tendency to have fewer comorbid medical conditions contribute to their improved prognosis compared with patients with ALI from other causes. Both groups of patients consume enormous amounts of hospital resources, and physicians and policy makers must be aware of this when future pandemics arise.

## Competing interests

The authors declare that they have no competing interests.

## Authors’ contributions

JW and JK were responsible for the data input. JW and JG composed the manuscript. HW provided editorial assistance. EW provided the statistical analysis. All authors read and approved the final manuscript.

## References

[B1] World now at the start of 2009 influenza pandemic 2009 Available: http://www.who.int/mediacentre/news/statements/2009/h1n1_pandemic_phase6_20090611/en/index.html [accessed 12/28, 2010]

[B2] Louie JK Acosta M Jamieson DJ Honein MA California Pandemic (H1N1) Working Group Severe 2009 H1N1 influenza in pregnant and postpartum women in California N Engl J Med 2010 362 27 35 10.1056/NEJMoa0910444 20032319

[B3] Libster R Bugna J Coviello S Pediatric hospitalizations associated with 2009 pandemic influenza A (H1N1) in Argentina N Engl J Med 2010 362 45 55 10.1056/NEJMoa0907673 20032320

[B4] Influenza Investigators ANZIC Webb SA Pettila V Critical care services and H1N1 influenza in Australia and New Zealand N Engl J Med 2009 2009 361 1925 1934 10.1056/NEJMoa0908481 19815860

[B5] Davies A Jones D Australia and New Zealand Extracorporeal Membrane Oxygenation (ANZ ECMO) Influenza Investigators Extracorporeal Membrane Oxygenation for 2009 Influenza A(H1N1) Acute Respiratory Distress Syndrome JAMA 2009 302 1888 1895 19822628 10.1001/jama.2009.1535

[B6] Dominguez-Cherit G Lapinsky SE Macias AE Critically Ill patients with 2009 influenza A(H1N1) in Mexico JAMA 2009 302 1880 1887 10.1001/jama.2009.1536 19822626

[B7] Rello J Rodriguez A Ibanez P Intensive care adult patients with severe respiratory failure caused by Influenza A (H1N1)v in Spain Crit Care 2009 13 R148 10.1186/cc8044 19747383 PMC2784367

[B8] Kumar A Zarychanski R Pinto R Critically ill patients with 2009 influenza A(H1N1) infection in Canada JAMA 2009 302 1872 1879 10.1001/jama.2009.1496 19822627

[B9] Narain JP Kumar R Bhatia R Pandemic (H1N1) 2009: epidemiological, clinical and prevention aspects Natl Med J India 2009 22 242 247 20334046

[B10] Jain S Kamimoto L Bramley AM Hospitalized patients with 2009 H1N1 influenza in the United States, April-June 2009 N Engl J Med 2009 361 1935 1944 10.1056/NEJMoa0906695 19815859

[B11] Patel M Dennis A Flutter C Khan Z Pandemic (H1N1) 2009 influenza Br J Anaesth 2010 104 128 142 10.1093/bja/aep375 20053625 PMC7094516

[B12] Bautista E Chotpitayasunondh T Writing Committee of the WHO Consultation on Clinical Aspects of Pandemic (H1N1) 2009 Influenza Clinical aspects of pandemic 2009 influenza A (H1N1) virus infection N Engl J Med 2010 362 1708 1719 20445182 10.1056/NEJMra1000449

[B13] Hui DS Lee N Chan PK Clinical management of pandemic 2009 influenza A(H1N1) infection Chest 2010 137 916 925 10.1378/chest.09-2344 20022969 PMC7094244

[B14] Ramsey C Kumar A H1N1: viral pneumonia as a cause of acute respiratory distress syndrome Curr Opin Crit Care 2010 17 64 71 21157318 10.1097/MCC.0b013e3283427259

[B15] Ramsey CD Funk D 3rd Miller RR Kumar A Ventilator management for hypoxemic respiratory failure attributable to H1N1 novel swine origin influenza virus Crit Care Med 2010 38 e58 65 20042855 10.1097/CCM.0b013e3181cde600

[B16] Venkata C Sampathkumar P Afessa B Hospitalized patients with 2009 H1N1 influenza infection: the Mayo Clinic experience Mayo Clin Proc 2010 85 798 805 10.4065/mcp.2010.0166 20664021 PMC2931615

[B17] Miller RR 3rd Markewitz BA Rolfs RT Clinical findings and demographic factors associated with ICU admission in Utah due to novel 2009 influenza A(H1N1) infection Chest 2010 137 752 758 10.1378/chest.09-2517 19933372 PMC3198489

[B18] Keogh-Brown MR Smith RD Edmunds JW Beutels P The macroeconomic impact of pandemic influenza: estimates from models of the United Kingdom, France, Belgium and The Netherlands Eur J Health Econ 2010 11 543 554 10.1007/s10198-009-0210-1 19997956

[B19] Dan YY Tambyah PA Sim J Cost-effectiveness analysis of hospital infection control response to an epidemic respiratory virus threat Emerg Infect Dis 2009 15 1909 1916 10.3201/eid1512.090902 19961669 PMC3044543

[B20] Brouwers L Cakici B Camitz M Tegnell A Boman M Economic consequences to society of pandemic H1N1 influenza 2009 - preliminary results for Sweden Euro Surveill 2009 14 19333 19761738 10.2807/ese.14.37.19333-en

[B21] Khazeni N Hutton DW Garber AM Hupert N Owens DK Effectiveness and cost-effectiveness of vaccination against pandemic influenza (H1N1) 2009 Ann Intern Med 2009 151 829 839 20008759 10.1059/0003-4819-151-12-200912150-00157 PMC3250217

[B22] Harris PA Taylor R Thielke R Payne J Gonzalez N Conde JG Research electronic data capture (REDCap)–a metadata-driven methodology and workflow process for providing translational research informatics support J Biomed Inform 2009 42 377 381 10.1016/j.jbi.2008.08.010 18929686 PMC2700030

[B23] Siau C Law J Tee A Poulose V Raghuram J Severe refractory hypoxaemia in H1N1 (2009) intensive care patients: initial experience in an Asian regional hospital Singapore Med J 2010 51 490 495 20658109

[B24] Gattinoni L Caironi P Cressoni M Lung recruitment in patients with the acute respiratory distress syndrome N Engl J Med 2006 354 1775 1786 10.1056/NEJMoa052052 16641394

[B25] Rubenfeld GD Herridge MS Epidemiology and outcomes of acute lung injury Chest 2007 131 554 562 10.1378/chest.06-1976 17296661

[B26] Amato MB Barbas CS Medeiros DM Effect of a protective-ventilation strategy on mortality in the acute respiratory distress syndrome N Engl J Med 1998 338 347 354 10.1056/NEJM199802053380602 9449727

[B27] Bellamy PE Oye RK Adult respiratory distress syndrome: hospital charges and outcome according to underlying disease Crit Care Med 1984 12 622 625 10.1097/00003246-198408000-00002 6744902

[B28] Rossi C Simini B Brazzi L Variable costs of ICU patients: a multicenter prospective study Intensive Care Med 2006 32 545 552 10.1007/s00134-006-0080-2 16501946

